# Further contributions to the Hydradephaga (Coleoptera, Haliplidae, Gyrinidae and Dytiscidae) fauna of Prince Edward Island, Canada: new records, distributions and faunal composition

**DOI:** 10.3897/zookeys.600.8856

**Published:** 2016-06-22

**Authors:** Yves Alarie

**Affiliations:** 1 Department of Biology, Laurentian University, Ramsey Lake Road, Sudbury, ON, Canada P3E 2C6

**Keywords:** Coleoptera, Maritime Ecozone, Prince Edward Island, Hydradephaga, faunistic, biodiversity

## Abstract

The Haliplidae, Gyrinidae and Dytiscidae (Coleoptera) of Prince Edward Island, Canada were surveyed during the years 2004–2005. A total of 2450 individuals from 79 species were collected from 98 different localities, among which 30 species are newly recorded from that region. Among these, *Acilius sylvanus* Hilsenhoff, *Rhantus consimilis* Motschulsky and *Neoporus sulcipennis* (Fall) stand out as representing the easternmost reports of these species in Canada. Once removed, *Gyrinus aquiris* LeConte (Gyrinidae) is reinstated in the faunal list of Prince Edward Island. According to this study and literature 84 species of Hydradephaga are currently known from Prince Edward Island. The Nearctic component of the fauna is made up of 68 species (80.9%) and the Holarctic component of 16 species (19.1%). Most species are characteristic of the Boreal and Atlantic Maritime Ecozones and have a transcontinental distribution. In an examination of the Hydradephaga of insular portions of Atlantic Canada, we found that despite significantly different land areas and different distances to the neighbouring continental mainland the island faunas of Prince Edward Island and insular Newfoundland are very similar in the number of species (84 and 94 species respectively) despite differences in composition. With a land area significantly larger than that of Prince Edward Island, however, the fauna of Cape Breton Island was 39% smaller consisting of 53 species. This difference could be due to the comparative lack of collecting efforts on Cape Breton Island.

## Introduction

The Maritime Provinces are a region of eastern Canada on the Atlantic coast consisting of New Brunswick, Nova Scotia, and Prince Edward Island. These provinces lie within the Atlantic Maritime Ecozone along with Québec’s Gaspé Peninsula, Magdalen Archipelago and portions of the south shore of the St. Lawrence River. The climate of this ecozone is strongly influenced by the Atlantic Ocean, which produces cooler summers (average 14 °C) and warmer winters (average -5 °C), with coastal areas having slightly warmer winters and cooler summers than inland. The Atlantic Ocean also provides moisture to the region, producing mean precipitation of 900 mm a year inland and 1500 mm a year on the coast. Geologically, this region is a mix of sedimentary and igneous bedrock (Alarie 2009).

Prince Edward Island is located at 46 degrees latitude, 63 degrees longitude in the Gulf of St. Lawrence, off the Atlantic Coast of the Canadian mainland. This crescent shaped Island is only 224 km long and between 6 km and 64 km wide and is separated from the mainland by the Northumberland Strait. Its total land mass is 5,656 sq. km. The island has many wetlands and rivers, most of which are quite small. Often wide and relatively shallow many of these rivers experience a tidal influence over much of their length. Extensive salt and freshwater wetlands are, therefore, associated with several of the larger rivers.

Aquatic Adephaga have many morphological adaptations to their aquatic environment, making them excellent subjects for ecological and biogeographic studies (Moreno et al. 1997). Additionally, these beetles are important indicators of spatial and temporal changes in the environment. This is why some authors have used them as bio-indicators of habitat quality in terms of nutrient enrichment or the presence of potential pollutants (e.g., Arnott et al. 2006; Sánchez-Fernández et al. 2006). These ecosystems, however, are highly vulnerable to threats related to intensive human influences (Della Bella et al. 2005), thereof the importance of conducting faunistic surveys to help at documenting the diversity of these beetles.

Investigations of the Dytiscidae, Haliplidae, and Gyrinidae of the Maritime Provinces have been sporadic and regionally variable. Recent papers (Majka 2008; Majka and Kenner 2009; Majka et al. 2009; Alarie 2009; Webster 2008; Webster and DeMerchant 2012; Webster et al. 2016) resulted in a better understanding of the Hydradephaga fauna in some areas. Despite many additions made recently by Majka (2008) the faunal list of Hydradephaga of Prince Edward Island is deemed incomplete (Majka 2008). This study aims to fill this gap by presenting for the first time the results of an extensive field oriented research on the Hydradephaga biodiversity of Prince Edward Island. It is conceivable that this study, complementing the previous works, should help to get a clearer picture of the diversity of Hydradephaga on this region.

## Methods and conventions

### Study areas

Geologically, Prince Edward Island is part of the ‘Maritimes Basin’, a geographically low area that was filled hundreds of millions of years ago by sandy sediments eroded from the newly formed Appalachian Mountains to the south and west. Prince Edward Island’s landscape has been largely influenced by the shape of the bedrock and by the ease with which it has been eroded. Low cliffs predominate along much of the shoreline especially on the northern headlands. The southern coastline, however, is more protected and erosion is, therefore, less pronounced. The several glaciers, which once covered Prince Edward Island, resulted in a nearly level to gently rolling landscape over much of the province. Approximately three-quarters of the land area is less than 50 meters above sea level, but a few hills throughout the central section of the Island have elevations of 150 meters (MacAlpine and Smith 2010).

Geographically, Prince Edward Island is subdivided into three counties: Kings, Prince, and Queens. Kings County is the province’s smallest, most rural and least-populated county. That region is also least dependent upon the agriculture industry compared with the other two counties, while being more heavily dependent on the fishery and forest industry. Prince County is located in western part of the island; its defining geographic feature is Malpeque Bay, a sub-basin of the Gulf of St. Lawrence, which creates the narrowest portion of Prince Edwards’s landmass. Much of Prince Edward Island’s industrial base is concentrated in the eastern part of that county. Lastly, Queens County, located in central Prince Edward Island. The county is geographically divided by the Hillsborough River’s estuary, a tidal inlet, which almost splits the county and Prince Edward Island. It is the richest and most populous county in the province. Queens County’s geography varies from picturesque shorelines of sandstone cliffs, sandy beaches and sheltered bays on the Gulf of St. Lawrence and Northumberland Straight, to extensive farming operations throughout interior regions. Topography ranges from relatively flat to rolling hills in the central hill lands known as the Bonshaw Hills.

### Collecting methods

Collections were conducted over three periods, May 15–19, 2004, September 19–24, 2004 and September 17–21, 2005, which essentially reflects a similar collecting effort in each county. Sampling was unstructured and qualitative with the goal of obtaining a strict inventory of Hydradephaga of Prince Edward Island. Beetles were collected using D-net sweeps in a variety of microhabitats including macrophyte beds, rocky shores, organic-rich sediments, and open water. Overall 98 samples were obtained, which are listed in Table 1, along with locality data and habitat information.

**Table 1.** Prince Edward Island (Canada) sampling localities and habitats (2004–2005); letter in sample code refers to the county (K = Kings; P = Prince; Q = Queens).

Sample	Locality	Habitat
01K	Kings Co. South of Dover. 17.ix.2005	River, flowing into Murray River; saline at level of Mt Pleasant; shoreline densely covered with *Lemna* sp.
02K	Kings Co. Hwy 325, 1 km North of Hwy 17. 17.ix.2005	Pools in *Picea* sp. forest; densely covered with vegetation
03K	Kings Co. Jct Hwy 325 & Hwy 202. 17.ix.2005	Creek
04K	Kings Co. Hwy 325 2 km N. of jct to Hwy 202. 17.ix.2005	Shallow pond on sandy bottom
05K	Kings Co. Hwy 316 off Hwy 17. 18.ix.2005	Roadside fen with abundance of *Equisetum* sp.; dark yellow water with heavy accumulation of organic matter
06K	Kings Co. Hwy 318 off Hwy 17a. 18.ix.2005	*Sphagnum* sp. bog; sampling along shoreline under mats of shrubs
07K	Kings Co. S. of Hwy 324. 18.ix.2005	Eutrophic creek with *Typha* sp. and shrubs
08K	Kings Co. Hwy 17 S. of Hwy 324. 18.ix.2005	Creek with swift currents
09K	Kings Co. Hwy 17 S. of Hwy 324. 18.ix.2005	Woodland pool with heavy accumulation of organic matter, mainly dead leaves
10Q	Queen’s Co. Off Hwy 4 near jct with Hwy 202. 18.ix.2005	*Sphagnum* sp. bog
11K	Kings Co. Hwy 2 near jct with Hwy 330. 19.ix.2005	Pond, shoreline with dense mats of Gramineae
12K	Kings Co. Chepstow at Hwy 16. 19.ix.2005	Small eutrophic creek in *Picea* sp. forest, slow moving water; samples from shallowest parts, under mats of dense vegetation
13K	Kings Co. Black Pond at Hwy 16, E. of Little Harbour. 19.ix.2005	Huge pond, located just beside sea
14K	Kings Co. MacVanes Creek at Hwy 16, east of Bothwell. 19.ix.2005	Eutrophic creek, almost still water
15K	Kings Co. Hwy 302 off Hwy 16. 19.ix.2005	Pond with *Nymphaea* sp.
16K	Kings Co. Hwy 302 off Hwy 16. 19.ix.2005	*Sphagnum* sp. bog; cold water; sampling under *Picea* sp. trees.
17K	Kings Co. Hwy 302 off Hwy 16. 19.ix.2005	Roadside ditch, at the edge of a bog; very dark brown water; presence of *Carex* sp. and *Typha* sp.
18K	Kings Co. Hwy 303 16 km W. of Hwy 16. 19.ix.2005	Fen; woodland pond with *Carex* sp. and Gramineae
19K	Kings Co. Hwy 303 12 km W. of Hwy 16. 19.ix.2005	Pond with *Nymphaea* sp.
20K	Kings Co. Hwy 306 3 km W. of Hwy 2. 20.ix.2005	Fen; densely covered with *Scirpus* sp.; moss along shoreline; slowly moving water; very dark brown water; *Acer rubrum*, *Picea* sp. and *Alnus* sp. forest
21K	Kings Co. Hwy 306 3 km W. of Hwy 2. 20.ix.2005	*Typha* sp. pond with abundance of *Equisetum* sp. in shallowest parts
22K	Kings Co. Hay River, W. of Clearspring at Hwy 6. 20.ix.2005	River with dark brown water; bed with large bolders
23K	Kings Co. Larkins Pond at Hwy 357, W. of Hwy 308. 20.ix.2005	Pond with clear water; shoreline with *Typha* sp.
24K	Kings Co. Goose River Road, off Hwy 16. 20.ix.2005	Small pond with mats of *Carex* sp.
25K	Kings Co. Cable Head, Schooner Creek, at Hwy 16. 20.ix.2005	Eutrophic creek with swift current; beetles collected underneath banks
26K	Kings Co. Cable Head W. at Hwy 336. 20.ix.2005	Wetland with *Typha* sp. along shoreline
27K	Kings Co. Cable Head W. at Hwy 336. 20.ix.2005	*Sphagnum* sp. bog; abundance of Ericaceae
28K	Kings Co. Schooner River at Hwy 336. 20.ix.2005	River with very dark water
29K	Kings Co. Hwy 337, 1 km off Hwy 313. 21.ix.2005	Wetlands; sampling in mats of *Calamagrostis* sp.
30K	Kings Co. Junction Hwy 313 & Hwy 321. 21.ix.2005	Wetland in *Picea* sp. forest; dense accumulation of *Calamagrostis* sp., *Juncus* sp. and *Typha* sp.
31K	Kings Co. Jct Hwy 321 close to Martinvale. 21.ix.2005	Wetlands
32K	Kings Co. Hwy 320 near jct Hwy 322. 21.ix.2005	*Carex* sp. pools in *Picea* sp. forest; bed with black sediments
33K	Kings Co. Cherry Hills at Hwy 351. 21.ix.2005	Eutrophic Creek
34P	Prince Co. Tignish at Hwy 153. 15.v.2004	Permanent pond on clay bottom; at edge of *Picea* sp. and *Populus* sp. forest
35P	Prince Co. Donohue Rd, off Hwy 12, near Fisherman’s Haven Provincial Park. 15.v.2004	Roadside ditch
36P	Prince Co. Green Mount at Hwy 162. 15.v.2004	*Typha* sp. pond
37P	Prince Co. Birch Groove Rd, off Hwy 153. 15.v.2004	Ephemeral woodland pool; dense accumulation of dead *Acer* sp. leaves; dark brown water
38P	Prince Co. Alberton, jct Hwy 150 and Hwy 12. 15.v.2004	Creek; heavily covered with *Carex* sp.
39P	Prince Co. Hwy 2, 1 km W. jct Hwy 151. 15.v.2004	Creek on sandy bed; sparse Gramineae along shoreline; very cold water
40P	Prince Co. Palmer Rd. 15.v.2004	Roadside woodland ditch, shallow, with heavy accumulation of organic debris; abundance of mosquito larvae
41P	Prince Co. Hwy 151, S. of Loretta. 16.v.2004	Creek, rocky bed, lacking vegetation
42P	Prince Co. Miminegash River at Hwy 151, near St. Lawrence. 16.v.2004	River
43P	Prince Co. Old Town Rd off Hwy 151. 16.v.2004	Roadside ditch; heavy accumulation of organic debris
44P	Prince Co. Old Town Rd off Hwy 151. 16.v.2004	Eutrophic creek; slow moving, dark brown water; heavy accumulation of organic debris
45P	Prince Co. Mill River at Hwy 148, near Howlan. 16.v.2004	River
46P	Prince Co. O’Leary, off Hwy 148. 16.v.2004	Shallow eutrophic creek
47P	Prince Co. Dublan, at Hwy 14. 16.v.2004	Pond; heavy accumulation of organic debris; dark brown water
48P	Prince Co. Hwy 142, near Roxberry. 17.v.2004	Permanent pond; heavy accumulation of *Sphagnum* sp., *Typha* sp., and *Scirpus* sp. along shoreline
49P	Prince Co. Hwy 137, off Hwy 142. 17.v.2004	Permanent *Sphagnum* sp. bog with *Ledum* sp., *Larix* sp., and *Carex* sp.; dark brown water
50P	Prince Co. Hwy 138, 1 km off Hwy 2. 17.v.2004	Eutrophic ditch; heavy accumulation of organic debris; dark brown water
51P	Prince Co. Portage at Hwy 12. 17.v.2004	*Sphagnum* sp. bog lake
52P	Prince Co. Troy Rd. off Hwy 12. 17.v.2004	Shallow pond with mats of sedges and *Scirpus* sp.
53P	Prince Co. Hwy 2, 2 km W. jct Hwy 12. 18.v.2004	Permanent pond
54P	Prince Co. Hwy 12, near jct Hwy 131. 18.v.2004	Shallow creek, about 1 m wide; sandy bed
55P	Prince Co. Hwy 127, off Hwy 2, near St. Hubert 18.v.2004	Shallow creek, about 1 m wide; in *Abies balsamea* and *Betula alleghaniensis* forest; dense mats of bryophytes
56P	Prince Co. Jct Hwy 124 and Hwy 129. 18.v.2004	Road side ditch in *Betula papyrifera*, *Abies balsamea* and *Populus tremuloides* forest; heavy accumulation of organic debris; brown water
57P	Prince Co. Jct Hwy 124 & Hwy 129. 18.v.2004	Shallow creek, about 15 cm deep
58P	Prince Co. Evangeline, off Hwy 11. 18.v.2004	Shallow ephemeral ditch
59P	Prince Co. Hwy 165, 1km off Hwy 11. 18.v.2004	Roadside ditch; abundance of *Equisetum* sp. and Gramineae in *Acer* sp., *Abies balsamea* and *Betula papyrifera* forest
60P	Prince Co. St. Nicholas, off Hwy 11. 19.v.2004	*Sphagnum* bog in *Picea* sp. forest; dense accumulation of *Carex* sp. and bryophytes; dark brown water
61P	Prince Co. St. Nicholas, 1 km N. Hwy 11. 19.v.2004	*Sphagnum* bog lake in *Abies balsamifera* forest
62P	Prince Co. Hwy 122 off Hwy 2. 19.v.2004	Permanent pond; shallowest sections of pond with mats of Gramineae and sparse *Typha* sp.
63P	Prince Co. Hwy 123 off Hwy 12. 19.v.2004	Eutrophic creek with mats of Gramineae; slow moving water
64P	Prince Co. North of Miscouche, off Hwy 12. 19.v.2004	Large *Typha* sp. pond
65P	Prince Co. Hwy 122. 19.v.2004.	Shallow eutrophic roadside ditch
66Q	Queens Co. Irishtown at Hwy 104. 19.ix.2004	Creek, rocky bed; swift moving water
67Q	Queens Co. Henry Craig ln, off Branders Pond Rd. 1 km off Hwy 20. 19.ix.2004	Shallow creek on sandy beach with sparse *Typha* sp.
68P	Prince Co. Bedeque, Dunk River at Hwy 171. 19.ix.2004	River, on clay bed
69P	Prince Co. Searletown, jct Hwy 111 and Hwy 112. 20.ix.2004	Shallow creek on clay bed; slow moving water; dense vegetation
70P	Prince Co. Augustine Cove, jct Hwy 10 and Hwy 117. 20.ix.2004	Salted marsh
71P	Prince Co. North Tyron Memorial Park, off Hwy 115 N. 20.ix.2004	Eutrophic creek; boulders covered with algae
72P	Prince Co. Hwy 16 near South Melville. 20.ix.2004	Roadside ditch in *Picea* sp. forest
73P	Prince Co. Hwy 16 near South Melville. 20.ix.2004	Shallow creek on rocky bed; swift flowing, cold and clear water
74P	Prince Co. Hwy 101, 2 km off Hwy 2. 21.ix.2004	Creek; swift flowing water; discharge of pond; dense accumulation of vegetation
75Q	Queens Co. Park Corner, Shining Lake at Hwy 20. 21.ix.2004	Lake shoreline with *Typha* sp.
76Q	Queens Co. Hwy 8, 2 km off Hwy 6. 21.ix.2004	Shallow pool with dense vegetation along shoreline
77P	Prince Co. Memorial Trail, Dunk River, at Hwy 109. 21.ix.2004	Small stream
78P	Prince Co. Wilmst River, at Hwy 109. 21.ix.2004	Small stream
79Q	Queens Co. Hwy 6 near jct Hwy 233. 21.ix.2004	Creek
80Q	Queens Co. Rollings Pond near North Rustico. 21.ix.2004	Deep *Typha* sp. pond
81Q	Queens Co. North Rustico. 21.ix.2004	Shallow pond covered with vegetation
82Q	Queens Co. Hwy 225 off Hwy 114. 22.ix.2004	Pond; muddy bed
83Q	Queens Co. Breadalbane at Hwy 231. 22.ix.2004	Deep *Typha* sp. pond
84Q	Queens Co. Hunter River, Hwy 13, near Hwy 251. 22.ix.2004	Eutrophic creek, muddy bed, with algae
85Q	Queens Co. Hwy 15, near Tracadie. 22.ix.2004	Shallow depression covered with vegetation
86Q	Queens Co. Hwy 15, near Tracadie. 22.ix.2004	Pond besides sand dunes; shoreline with *Scirpus* sp. and *Typha* sp.
87Q	Queens Co. Hwy 15, near Tracadie. 22.ix.2004	Ericaceae and *Carex* sp. plain
88Q	Queens Co. Hwy 222, near Pleasant Groove. 22.ix.2004	Roadside ditch with *Scirpus* sp. and Gramineae
89Q	Queens Co. Canoe Cove at Hwy 19. 23.ix.2004	Shallow creek covered with vegetation
90Q	Queens Co. Hwy 19, 2 km W. Cumberland. 23.ix.2004	Shallow creek on rocky bed; mats of Gramineae; swift current
91Q	Queens Co. Bonshaw, off Hwy 1. 23.ix.2004	Pond
92Q	Queens Co. Green Bay Rd., off Hwy 237. 23.ix.2004	Small stream
93Q	Queens Co. Winsloe South, jct Hwy 223 and Hwy 256. 23.ix.2004	Shallow creek on rocky bed with dense vegetation
94Q	Queens Co. Winter River at Hwy 222. 23.ix.2004	River; slow moving water; dense vegetation along shoreline
95Q	Queens Co. Hwy 19 near Tracadie. 23.ix.2004	Creek; cold water with dense vegetation
96Q	Queens Co. Jct Hwy 271 and Hwy 218. 24.ix.2004	Marsh with *Alnus rugosa* and *Equisetum* sp. in *Picea* sp. forest
97Q	Queens Co. French Village, jct Hwy 217 and Hwy 218. 24.ix.2004	Marsh with *Scirpus* sp. and Ericaceae; dark brown water
98K	Kings Co. Hwy 320 off Hwy 22. 24.ix.2004	Small stream on rocky bed; abundance of *Alnus rugosa* along shoreline

### Nomenclature

Nomenclature is based on the classification in Oygur and Wolfe (1991) (Gyrinidae: *Gyrinus* Müller), Vondel (2005) (Haliplidae), Nilsson (2015) (Dytiscidae) and Gustafson and Miller (2015) (Gyrinidae: *Dineutus* MacLeay).

### Depositories

Voucher specimens are deposited in the author’s research collection (Department of Biology, Laurentian University, Sudbury, Ontario).

## Results

A total of 2450 specimens representing 79 species of Hydradephaga (9 Gyrinidae; 6 Haliplidae; 64 Dytiscidae) were collected in this study (Table 2). Among these, 30 species are reported for the first time and an additional one is reinstated in the faunal list of Prince Edward Island.

**Table 2.** Species of Hydradephaga (Dytiscidae, Gyrinidae, Haliplidae) collected in Prince Edward Island, Canada in 2004 and 2005 with sample numbers (as in Table 1), absolute (AF) and relative frequencies (%), and relative frequency of occurrence (RFO). Species and counties in bold denote new records from Prince Edward Island given in the present account.

Taxon	Sample numbers	AF (%)	RFO
**Gyrinidae**			
*Dineutus nigrior* Roberts	19K, 47P, 48P, 49P, 61P, 62P	32 (1.31)	0.06
*Gyrinus affinis* Aubé	02K, 34P, 47P, 49P, 50P, 56P, 61P, 79Q, 94Q	21 (0.86)	0.09
***Gyrinus aquiris* LeConte**	08K, 14K, 34P, 35P, 49P, 75Q, 78P, 87Q, 94Q, 95Q	147 (6.00)	0.10
*Gyrinus bifarius* Fall	28K	4 (0.16)	0.01
*Gyrinus confinis* Fall	13K, 33K, 75Q, 94Q	24 (0.98)	0.04
***Gyrinus latilimbus* Fall**	31K, 41P, 49P, 50P	5 (0.20)	0.04
*Gyrinus lecontei* (Hope)	02K, 28K, 34P, 50P, 56P, 61P, 62P, 63P, 65P, 79Q, 92Q, 98K	22 (0.90)	0.12
***Gyrinus pugionis* Fall**	31K, 38P, 47P, 49P	8 (0.33)	0.04
*Gyrinus sayi* Aubé	02K, 09K, 23K, 38P, 47P, 49P, 56P, 61P, 64P, 75Q, 79Q, 81Q, 87Q, 94Q, 95Q	61 (2.49)	0.15
**Haliplidae**			
*Haliplus canadensis* Wallis	48P	1 (0.04)	0.01
***Haliplus connexus* Matheson**	19K, 35P, 88Q	5 (0.20)	0.03
*Haliplus cribarius* LeConte	56P	1 (0.04)	0.01
*Haliplus immaculicollis* Harris	01K, 02K, 03K, 11K, 13K, 14K; 15K; 20K; 23K, 24K; 30K; 32K, 35P, 36P, 38P, 39P, 40P, 43P, 44P, 48P, 49P, 50P, 53P, 56P, 57P, 61P, 62P, 67Q, 71P, 73P, 76Q, 77P, 78P, 79Q, 81Q, 82Q, 83Q, 84Q, 87Q, 88Q, 91Q, 92Q, 93Q, 94Q, 98K	272 (11.10)	0.46
*Haliplus longulus* LeConte	35P, 37P, 49P, 65P, 02K, 05K, 06K, 17K	32 (0.13)	0.08
*Peltodytes edentulus* (LeConte)	39P, 45P, 56P, 67Q, 76Q	15 (0.61)	0.05
*Peltodytes tortulosus* Roberts	44P, 53P, 61P, 75Q, 02K	12 (0.49)	0.05
**Dytiscidae**			
*Acilius mediatus* (Say)	02K, 12K, 24K, 35P, 43P, 76Q	13 (0.53)	0.06
*Acilius semisulcatus* Aubé	09K, 15K, 24K, 27K, 32K, 34P, 43P, 50P, 52P, 62P, 78P, 81Q, 82Q, 88Q, 97Q	24 (0.98)	0.15
***Acilius sylvanus* Hilsenhoff**	34P	1 (0.04)	0.01
*Agabus ambiguus* (Say)	02K, 07K, 09K, 10Q, 12K, 13K, 17K, 18K, 19K, 27K, 32K, 35P, 36P, 38P, 46P, 55P, 62P, 67Q, 71P, 81Q, 82Q	60 (2.45)	0.21
*Agabus anthracinus* Mannerheim	01K, 02K, 05K, 06K, 07K, 09K, 10Q, 17K, 18K, 27K, 34P, 35P, 36P, 38P, 48P, 50P, 52P, 56P, 61P, 62P, 63P, 85Q, 87Q	87 (3.55)	0.24
***Agabus bifarius* (Kirby)**	06K, 27K, 32K, 52P	4 (0.16)	0.04
***Agabus erytropterus* (Say)**	02K, 11K, 12K, 31K, 32K	8 (0.33)	0.05
*Agabus phaeopterus* (Kirby)	38P	1 (0.04)	0.01
***Agabus punctulatus* Aubé**	34P	9 (0.37)	0.09
***Agabus semipunctatus* (Kirby)**	06K, 20K, 27K, 29K, 32K, 62P	9 (0.37)	0.06
*Agabus subfuscatus* Sharp	06K, 17K, 27K, 32K, 35P, 55P, 96Q	15 (0.61)	0.07
*Boreonectes griseostriatus* (DeGeer)	01K, 34P, 35P	10 (0.41)	0.03
***Colymbetes paykulli* Erichson**	27K, 76Q	2 (0.08)	0.02
*Colymbetes sculptilis* Harris	09K, 18K, 20K, 21K, 26K, 27K, 34P, 73P, 78P	14 (0.57)	0.09
*Copelatus glyphicus* (Say)	32K	2 (0.08)	0.01
*Coptotomus lenticus lenticus* LeConte	11K, 26K, 47P, 49P, 61P, 62P, 87Q, 97Q	12 (0.49)	0.08
*Desmopachria convexa* (Aubé)	02K, 05K, 27K, 43P, 44P, 48P, 52P, 60P, 97Q	27 (1.10)	0.09
*Dytiscus dauricus* Gebler	11K, 12K, 52P, 71P	4 (0.16)	0.04
*Dytiscus harisii* Kirby	51P	1 (0.04)	0.01
*Dytiscus verticalis* Say	17K, 18K, 49P, 56P, 62P, 84Q	6 (0.25)	0.06
***Graphoderus liberus* (Say)**	49P, 61P	2 (0.08)	0.02
*Graphoderus perplexus* Sharp	61P, 97Q	3 (0.12)	0.02
*Hydaticus aruspex* Clark	09K, 27K, 35P, 37P, 60P	6 (0.25)	0.05
***Hydrocolus paugus* (Fall)**	07K, 20K, 27K, 36P, 44P, 54P, 72P, 90Q, 98K	11 (0.45)	0.09
*Hydrocolus stagnalis* (Gemminger & Harold)	40P, 60P	2 (0.08)	0.02
*Hydroporus dentellus* Fall	02K, 04K, 05K, 06K, 15K, 27K	14 (0.57)	0.06
***Hydroporus fuscipennis* Schaum**	21K, 23K, 27K, 32K, 34P, 35P, 36P, 40P	27 (1.10)	0.08
***Hydroporus gossei* Larson & Roughley**	02K, 05K, 06K, 07K, 27K, 48P, 63P	21 (0.86)	0.07
*Hydroporus niger* Say	01K, 02K, 04K, 05K, 09K, 11K, 15K, 17K, 18K, 19K, 20K, 21K, 25K, 27K, 30K, 32K, 33K, 34P, 40P, 43P, 48P, 49P, 65P, 74P, 76Q, 88Q, 91Q, 97Q, 98K	97 (3.96)	0.30
*Hydroporus notabilis* LeConte	68P, 70P	2 (0.08)	0.02
***Hydroporus obscurus* Sturm**	10Q, 11K	16 (0.65)	0.02
*Hydroporus signatus* Mannerheim	02K, 05K, 09K, 15K, 17K, 18K, 20K, 23K, 24K, 32K, 34P, 35P, 40P, 49P	37 (1.51)	0.14
***Hydroporus striola* (Gyllenhal)**	02K, 05K, 06K, 09K, 11K, 12K, 13K, 15K, 18K, 20K, 21K, 27K, 29K, 30K, 32K, 37P, 40P, 48P, 49P, 52P, 58P, 59P, 60P, 72P, 81Q, 88Q	104 (4.25)	0.27
***Hydroporus tenebrosus* LeConte**	01K, 04K, 13K, 18K, 20K, 21K, 27K, 28K, 35P, 36P, 40P, 52P, 58P, 81Q, 88Q	39 (1.59)	0.15
***Hydroporus tristis* (Paykull)**	02K, 05K, 06K, 09K, 10Q, 15K, 21K, 25K, 27K, 32K, 35P, 40P, 44P, 60P	22 (0.90)	0.14
***Hygrotus compar* Fall**	34P, 35P	6 (0.25)	0.02
*Hygrotus impressopunctatus* (Schaller)	09K, 34P, 35P, 36P, 60P, 68P, 76Q	29 (1.18)	0.07
***Hygrotus laccophilinus* (LeConte)**	02K, 05K, 06K, 27K, 29K, 30K, 50P, 62P, 88Q	22 (0.90)	0.09
*Hygrotus picatus* (Kirby)	06K, 09K, 17K, 27K, 30K, 31K, 32K, 36P, 52P, 60P	27 (1.10)	0.10
*Hygrotus sayi* Balfour-Browne	02K, 05K, 09K, 11K, 13K, 14K, 15K, 21K, 23K, 26K, 27K, 36P, 37P, 44P, 48P, 49P, 50P, 51P, 52P, 53P, 56P, 61P, 62P, 67Q, 75Q, 76Q, 80Q, 81Q, 83Q, 88Q, 94Q	76 (3.10)	0.32
*Hygrotus turbidus* (LeConte)	09K, 17K, 18K, 27K, 35P, 50P, 56P, 62P	17 (0.69)	0.08
*Ilybiosoma seriatum* (Say)	08K, 12K, 16K, 38P, 39P, 41P, 44P, 46P, 54P, 55P, 69P, 71P, 73P, 76Q, 77P, 89Q, 98K	77 (3.14)	0.17
*Ilybius angustior* (Gyllenhal)	15K	1 (0.04)	0.01
*Ilybius biguttulus* (Germar)	01K, 02K, 04K, 05K, 12K, 13K, 15K, 17K, 19K, 23K, 24K, 27K, 30K, 31K, 44P, 48P, 50P, 56P, 68P, 75Q, 76Q, 81Q, 88Q, 91Q	67 (2.73)	0.25
***Ilybius discedens*** Sharp	07K, 10Q, 20K, 27K, 51P, 88Q	7 (0.29)	0.06
***Ilybius erichsoni* Gemminger & Harold**	34P, 37P, 57P	5 (0.20)	0.03
***Ilybius larsoni* (Fery & Nilsson)**	07K, 10Q, 20K, 27K, 88Q	6 (0.25)	0.05
*Ilybius pleuriticus* (LeConte)	80Q, 83Q	2 (0.08)	0.02
*Laccophilus maculosus maculosus* Say	04K, 09K, 11K, 13K, 16K, 19K, 20K, 23K, 24K, 34P, 35P, 36P, 38P, 44P, 47P, 48P, 49P, 51P, 56P, 61P, 62P, 75Q, 76Q, 80Q, 81Q, 83Q, 88Q, 94Q, 97Q	69 (2.82)	0.30
***Laccornis latens* (Fall)**	20K	2 (0.08)	0.01
*Liodessus affinis* (Say)	34P, 40P, 48P, 56P, 62P, 76Q, 78P, 88Q, 97Q	15 (0.61)	0.09
*Nebrioporus rotundatus* (LeConte)	01K, 14K, 22K, 28K, 29K, 38P, 42P, 45P, 64P, 66Q, 67Q, 71P, 74P, 76Q, 77P, 78P, 79Q, 83Q, 84Q, 92Q, 93Q, 94Q, 98K	162 (6.61)	0.24
***Neoporus carolinus* (Fall)**	44P, 50P, 55P, 56P, 57P, 76Q	49 (2.00)	0.06
***Neoporus clypealis* (Sharp)**	01K, 11K, 14K, 30K, 31K, 44P, 92Q, 93Q, 98K	22 (0.90)	0.09
*Neoporus dimidiatus* (Gemminger & Harold)	01K, 11K, 23K, 38P, 42P, 45P, 66Q, 71P, 74P, 77P, 78P, 79Q, 80Q, 81Q, 83Q, 93Q, 94Q, 98K	114 (4.65)	0.18
***Neoporus sulcipennis* (Fall)**	42P, 45P	28 (1.14)	0.02
*Neoporus undulatus* (Say)	01K, 09K, 11K, 13K, 15K, 18K, 20K, 21K, 23K, 24K, 25K, 26K, 27K, 30K, 31K, 32K, 36P, 38P, 42P, 47P, 48P, 53P, 56P, 64P, 65P, 67Q, 75Q, 76Q, 78P, 80Q, 81Q, 83Q, 84Q, 86Q, 87Q, 88Q, 94Q	227 (9.27)	0.38
*Rhantus binotatus* (Harris)	09K, 15K, 20K, 24K, 26K, 35P, 36P, 44P, 48P, 49P, 62P, 72P, 76Q, 81Q, 83Q, 94Q	30 (1.23)	0.16
***Rhantus consimilis* Motschulsky**	34P	5 (0.20)	0.01
***Rhantus sinuatus* (LeConte)**	15K, 27K	5 (0.20)	0.02
***Rhantus suturellus* (Harris)**	32K	1 (0.04)	0.01
*Rhantus wallisi* Hatch	53P, 61P	4 (0.16)	0.02
***Sanfillipodytes pseudovilis* (Young)**	54P	1 (0.20)	0.01
	Total	2450	

### Faunistic and bionomics notes on the species newly collected from the Province of Prince Edward Island (Canada)

#### GYRINIDAE

##### Gyrinus aquiris

Taxon classificationAnimaliaColeopteraGyrinidae

LeConte

###### Note.

*Gyrinus aquiris* was the most abundant (45.4%) and one of the most frequently collected gyrinid (6%) in this survey. The species was collected in every county of Prince Edward Island (Table 2).

**Table 3.** Checklist of species of Hydradephaga recorded from Prince Edward Island, Canada, and their provincial and territorial distribution within northeastern North America (NA). *, Holarctic species; †, species not collected in this survey but recorded in Majka (2008); K, Kings County; P, Prince County; Q, Queens County.

Taxon	Counties			Distribution in northeastern NA
	P	Q	K	
**GYRINIDAE**				
**Gyrininae**				
Dineutini				
*Dineutus assimilis* Kirby†		1		CT, ME, NB, NS, ON, PE, QC, RI
*Dineutus hornii* Roberts†		1	1	CT, MA, ME, MI, NB, NH, NS, NY, ON, PE, QC, RI
*Dineutus nigrior* Roberts	1		1	CT, MA, ME, MI, NB, NH, NS, ON, PE, QC, RI
Gyrinini				
*Gyrinus affinis* Aubé	1	1	1	LB, MA, ME, NB, NF, NH, NS, NY, ON, PE, QC, RI, VT
*Gyrinus aquiris* LeConte	1	1	1	LB, MA, ME, MI, NB, NF, NS, NY, ON, PE, QC, RI
*Gyrinus bifarius* Fall	1	1	1	LB, MA, ME, NB, NF, NH, NS, NY, ON, PE, QC
*Gyrinus confinis* Fall		1	1	CT, LB, MA, ME, NB, NF, NH, NS, NY, ON, PE, QC, SM, VT
*Gyrinus fraternus* Couper†		1		MA, ME, NB, NH, NS, NY, ON, PE, QC, VT
*Gyrinus latilimbus* Fall	1		1	CT, LB, MA, ME, NB, NF, NH, NS, NY, ON, PE, QC, SE
*Gyrinus lecontei* (Hope)	1	1	1	CT, MA, ME, NB, NH, NS, NY, ON, PE, QC, RI
*Gyrinus pugionis* Fall	1		1	MA, ME, MI, NB, NH, NS, NY, ON, PE, QC. VT
*Gyrinus sayi* Aubé	1	1	1	CT, MI, LB, MA, ME, NB, NF, NH, NS, NY, ON, PE, QC, RI, SM
**HALIPLIDAE**				
*Haliplus canadensis* Wallis	1		1	MA, NB, NS, ON, PE, QC
*Haliplus connexus* Matheson	1	1	1	CT, MA, ME, NB, NH, NS, NY, ON, PE, QC, VT
*Haliplus cribarius* LeConte	1	1	1	CT, LB, MA, ME, MI, NB, NF, NH, NS, NY, ON, PE, QC, SM
*Haliplus immaculicollis* Harris	1	1	1	CT, LB, MA, ME, MI, NB, NF, NH, NS, NY, ON, QC, PE, RI, SM, VT
*Haliplus longulus* LeConte	1	1	1	MA, ME, NB, NH, NB, NS, NY, ON, PE, QC, RI
*Peltodytes edentulus* (LeConte)	1	1		MA, NB, NH, NS, ON, QC, PE, RI
*Peltodytes tortulosus* Roberts	1	1	1	ME, NB, NH, NS, NY, ON, PE, QC
**DYTISCIDAE**				
Agabinae				
Agabini				
*Agabus ambiguus* (Say)	1	1	1	LB, ME, MI, NB, NF, NH, NS, ON, PE, QC, RI, SM
*Agabus anthracinus* Mannerheim	1	1	1	LB, MA, ME, MI, NB, NF, NH, NS, NY, ON, PE, QC, SM, VT
*Agabus bifarius* (Kirby)*	1		1	LB, MA, ME, NB, NH, NS, NY, ON, PE,QC, RI
*Agabus discolor* (Harris)* †		1		LB, MA, ME, NB, NH, NS, NY, ON, PE, QC, VT
*Agabus erytropterus* (Say)			1	CT, LB, MA, ME, NB, NF, NS, NY, ON, PE, QC, RI
*Agabus phaeopterus* (Kirby)	1	1		LB, MA, ME, MI, NB, NF, NH, NS, NY, ON, PE, QC
*Agabus punctulatus* Aubé	1			LB, MA, ME, NB, NH, NS, ON, PE, QC, RI
*Agabus semipunctatus* (Kirby)	1		1	CT, LB, MA, ME, MI, NB, NF, NH, NS, NY, ON, QC, RI
*Agabus subfuscatus* Sharp	1	1	1	CT, LB, MA, ME, NB, NF, NH, NS, ON, PE, QC, VT
*Ilybiosoma seriatum* (Say)	1	1	1	CT, LB, MA, ME, MI, NB, NF, NH, NS, ON, PE, QC, SM
*Ilybius angustior* (Gyllenhal)*		1	1	LB, MI, ME, NB, NF, NH, NS, ON, PE, QC, SM, VT
*Ilybius biguttulus* (Germar)	1	1	1	MA, ME, MI, NB, NF, NH, NS, NY, ON, PE, QC, RI, SM, VT
*Ilybius discedens* Sharp	1	1	1	LB, ME, MI, NB, NF, NH, NS, ON, PE, QC, SM
*Ilybius erichsoni* Gemminger & Harold*	1			LB, MA, ME, MI, NB, NF, NH, NS, NY, ON, PE, QC
*Ilybius larsoni* (Fery & Nilsson)		1	1	NB, NS, ON, PE, QC
*Ilybius pleuriticus* (LeConte)		1		CT, LB, MA, ME, MI, NB, NF, NS, ON, PE, QC, RI, SM, VT
Colymbetinae				
Colymbetini				
*Colymbetes paykulli* Erichson*		1	1	LB, ME, NB, NF, NS, ON, PE, QC
*Colymbetes sculptilis* Harris	1	1	1	CT, LB, MI, NB, NF, NH, NS, NY, ON, PE, QC, RI
*Rhantus binotatus* (Harris)	1	1	1	CT, LB, ME, MI, NB, NF, NH, NS, ON, PE, QC, RI, SM
*Rhantus consimilis* Motschulsky	1			MA, ME, NB, NH, NY, PE, QC, VT
*Rhantus sinuatus* (LeConte)			1	LB, MA, ME, NB, NF, NH, NS, NY, ON, PE, QC
*Rhantus suturellus* (Harris)*			1	CT, LB, MA, ME, MI, NB, NF, NH, NS, NY, ON, PE, QC, SM
*Rhantus wallisi* Hatch	1	1		LB, MA, MI, NB, NF, NH, NS, ON, PE, QC, SM
Copelatinae				
Copelatini				
*Copelatus glyphicus* (Say)			1	CT, ME, NF, NH, NB, NS, ON, PE, QC, RI
Coptotominae				
Coptotomini				
*Coptotomus lenticus lenticus* LeConte	1	1	1	MA, ME, MI, NB, NH, NS, NY, ON, QC, NB, PE, RI
Dytiscinae				
Aciliini				
*Acilius mediatus* (Say)	1	1	1	CT, MA, NB, NH, NS, ON, PE, QC, RI
*Acilius semisulcatus* Aubé	1	1	1	CT, LB, MA, ME, MI, NB, NF, NH, NS, ON, PE, QC, RI, SM
*Acilius sylvanus* Hilsenhoff	1			MA, ME, NB, NH, NY, ON, PE, QC
*Graphoderus liberus* (Say)	1	1		CT, MA, ME, NB, NF, NH, NS, ON, PE, QC, RI, SM
*Graphoderus perplexus* Sharp*	1		1	LB, MA, ME, NB, NF, NH, NS, ON, PE, QC, SM
Dytiscini				
*Dytiscus dauricus* Gebler*	1	1	1	LB, ME, MI, NB, NF, NS, NY, ON, PE, QC, SM
*Dytiscus fasciventris* Say†			1	CT, LB, ME, NB, NH, NS, ON, PE, QC, RI
*Dytiscus harrisii* Kirby	1	1		CT, LB, ME, NB, NF, NH, NS, NY, ON, PE, QC
*Dytiscus verticalis* Say	1	1	1	CT, MA, ME, NB, NH, NS, NY, ON, PE, QC, RI
Hydaticini				
*Hydaticus aruspex* Clark*	1		1	CT, LB, MA, ME, NB, NF, NH, NS, ON, PE, QC
Hydroporinae				
Bidessini				
*Liodessus affinis* (Say)	1	1	1	CT, ME, NB, NF, NH, NS, ON, PE, QC, RI
Hydroporini				
*Boreonectes griseostriatus* (DeGeer)*	1		1	LB, ME, MI, NB, NF, NS, ON, PE, QC, RI, SM
*Hydrocolus paugus* (Fall)	1	1	1	LB, MA, ME, MI, NB, NF, NH, NS, NY, ON, PE, QC, SM
*Hydrocolus stagnalis* (Gemminger & Harold)	1	1	1	CT, MA, ME, NB, NH, NS, NY, ON, PE, QC
*Hydroporus dentellus* Fall		1	1	LB, MA, ME, NB, NH, NS, NY, ON, PE, QC
*Hydroporus fuscipennis* Schaum*	1		1	LB, MA, NB, NF, ON, PE, QC, RI
*Hydroporus gossei* Larson & Roughley	1		1	ME, NB, NF, NY, ON, PE, QC
*Hydroporus niger* Say	1	1	1	CT, MA, MI, NB, NF, NH, NS, NY, ON, PE, QC, RI
*Hydroporus notabilis* LeConte*	1	1		LB, MA, ME, MI, NB, NF, NH, NS, ON, PE, QC, SM
*Hydroporus obscurus* Sturm*		1	1	LB, NB, NF, NS, ON, PE, QC, SM
*Hydroporus signatus* Mannerheim	1		1	CT, LB, MA, ME, MI, NB, NF, NH, NS, NY, ON, PE, QC, RI, SM
*Hydroporus striola* (Gyllenhal)*	1	1	1	LB, ME, MI, NB, NF, NH, NS, ON, PE, QC, RI
*Hydroporus tenebrosus* LeConte	1	1	1	MA, ME, MI, NB, NH, NS, NF, ON, PE, QC,
*Hydroporus tristis* (Paykull)*	1		1	LB, ME, MI, NB, NF, NH, NY, ON, PE, QC, RI, SM
*Nebrioporus rotundatus* (LeConte)	1	1	1	LB, MA, ME, NB, NF, NS, ON, PE, QC, RI
*Neoporus carolinus* (Fall)	1	1		LB, MA, ME, NB, NF, NH, NS, NY, ON, PE, QC, SM
*Neoporus clypealis* (Sharp)	1	1	1	ME, NB, NH, NS, NY, ON, PE, QC
*Neoporus dimidiatus* (Gemminger & Harold)	1	1	1	CT, LB, MA, ME, NB, NF, NH, NS, ON, PE, QC, RI
*Neoporus sulcipennis* (Fall)	1			NB, NH, NY, ON, PE, QC,
*Neoporus undulatus* (Say)	1	1	1	CT, LB, MA, ME, MI, NB, NF, NS, NY, ON, PE, QC, RI, SM
*Sanfilippodytes pseudovilis* (Young)	1			LB, MI, NB, NF, ON, PE, QC
Hygrotini				
*Hygrotus compar* Fall	1			LB, ME, NB, NF, NH, ON, PE, QC
*Hygrotus impressopunctatus* (Schaller)*	1	1	1	LB, ME, MI, NB, NF, NH, NS, ON, PE, QC
*Hygrotus laccophilinus* (LeConte)	1	1	1	ME, NB, NH, NS, ON, PE, QC
*Hygrotus picatus* (Kirby)	1	1	1	LB, MI, NB, NF, NS, NY, ON, PE, QC, RI
*Hygrotus sayi* Balfour-Browne	1	1	1	LB, MA, ME, MI, NB, NF, NH, NS, NY, ON, PE, QC, RI, SM, VT
*Hygrotus turbidus* (LeConte)	1	1	1	MA, MI, NB, NH, NS, NY, ON, PE, QC, RI
Hyphydrini				
*Desmopachria convexa* (Aubé)	1	1	1	ME, MI, NB, NS, ON, PE, QC, RI
Laccornini				
*Laccornis latens* (Fall)			1	MA, NB, NH, NS, NY, ON, PE, QC,
Laccophilinae				
Laccophilini				
*Laccophilus maculosus maculosus* Say	1	1	1	CT, MA, ME, MI, NB, NH, NS, ON, PE, QC, RI
Totals	67	58	66	

**Notes**: Distributional information is derived from Downie and Arnett (1996), Larson et al. (2000), Majka (2008), Webster (2008), Alarie (2009), Majka et al. (2011), Elder and Abraham (2012), Bousquet et al. (2013), Webster and DeMerchant (2012), and Webster (2016). Regional Distribution: CT , Connecticut ; LB , Labrador ; MA , Massachusetts ; ME , Maine ; MI , Magdalen Island Archipelago ; NB , New Brunswick ; NF , insular Newfoundland ; NH , New Hampshire ; NS , Nova Scotia ; NY , New York ; ON , Ontario ; PE , Prince Edward Island ; QC , Québec ; RI , Rhode Island ; SM , Saint-Pierre et Miquelon ; VT , Vermont .

###### Habitat.

In Prince Edward Island, *Gyrinus aquiris* was collected both from lotic (50%) and lentic (50%) environments (Tables 1–2), which is similar to the observations made by Alarie (2009) in the Magdalen Islands. Oygur and Wolfe (1991), however, reported 80% of specimens from lentic habitats whereas Morrissette (1979) reported the species from ponds and lakes invaded by aquatic plants.

###### Distribution in the Maritime Ecozone.

*Gyrinus aquiris* was reported from the Magdalen Islands (Alarie 2009), and the Maritime Provinces of New Brunswick (Oygur and Wolfe 1991; Roughley 1991a; Webster 2016) and Nova Scotia (Campbell et al. 1987). Although Roughley (1991a) reported it from Prince Edward Island, Majka (2008) removed it from the province’s faunal list since there were no voucher specimens or published records.

##### Gyrinus latilimbus

Taxon classificationAnimaliaColeopteraGyrinidae

Fall

###### Note.

This very small gyrinid was collected in relatively low number (five specimens) at four different localities of Kings and Prince Counties (Table 2).

###### Habitat.

Robert (1955) and Morrissette (1979) reported this species from small deep lakes near dense emergent vegetation (i.e., *Carex* and *Scirpus* spp.). Oygur and Wolfe (1991) reported it primarily (71%) from lentic habitats. The specimens collected in this survey were predominantly from lentic habitats (Tables 1–2).

###### Distribution in the Maritime Ecozone.

*Gyrinus latilimbus* was reported from New Brunswick (Roughley 1991a; Webster 2016) and Nova Scotia (Roughley 1991a).

##### Gyrinus pugionis

Taxon classificationAnimaliaColeopteraGyrinidae

Fall

###### Note.

Eight specimens were collected from four different localities of Kings and Prince Counties (Table 2).

###### Habitat.

Robert (1955), Morrissette (1979) and Alarie (2009) reported *Gyrinus pugionis* from peaty or semi-peaty lakes; Robert (1955) mentioned that it invades narrow outflow streams from such lakes. Oygur and Wolfe (1991) reported it primarily (88%) from lentic habitats. In Prince Edward Island, most specimens were collected from lentic habitats (Tables 1–2).

###### Distribution in the Maritime Ecozone.

This species was reported from the Provinces of New Brunswick (Roughley 1991a; Webster 2016) and Nova Scotia (Roughley 1991a), and from the Magdalen Islands (Alarie 2009).

#### HALIPLIDAE

##### Haliplus connexus

Taxon classificationAnimaliaColeopteraHaliplidae

Matheson

###### Note.

Whereas found in relatively low numbers (5 specimens) *Haliplus connexus* is reported for in each of the three Prince Edward Island counties (Table 2).

###### Habitat.

In Atlantic Canada, there are records from small streams, eutrophic ponds, river margins, slow streams and temporary habitats (Majka et al. 2009). In Prince Edward Island, all specimens of *Haliplus connexus* were collected along the edge of weedy lentic habitats (Tables 1–2).

###### Distribution in the Maritime Ecozone.

The species is known also from the neighboring Maritime Provinces of New Brunswick and Nova Scotia (Matheson 1912; Wallis 1933; Roughley 1991b).

#### DYTISCIDAE

##### Acilius sylvanus

Taxon classificationAnimaliaColeopteraDytiscidae

Hilsenhoff

###### Note.

This species is known from Prince Edward Island by a single specimen record from Prince County (Table 2).

###### Habitat.

These beetles occur in the emergent zone of sun-warmed permanent or semi-permanent ponds in forested area (Larson et al. 2000). In Prince Edward Island, *Acilius sylvanus* was collected in a permanent pond on clay bottom located at the edge of a spruce and aspen forest (Tables 1).

###### Distribution in the Maritime Ecozone.

This species is known only from the neighboring Province of New Brunswick (Webster 2008). The presence of *Acilius sylvanus* in Prince Edward Island represents the easternmost record of this species in Canada.

##### Agabus bifarius

Taxon classificationAnimaliaColeopteraDytiscidae

(Kirby)

###### Note.

This species is reported from four specimens collected in Kings (3) and Prince Counties (1) (Table 2).

###### Habitat.

*Agabus bifarius* occurs amongst emergent grasses and sedges at the margin of a variety of lentic habitats, but it is especially characteristic of the margins of shallow, exposed, vernal ponds (Larson et al. 2000), which is essentially the type of habitats where it was found in Prince Edward Island (Table 1).

###### Distribution in the Maritime Ecozone.

This species is also known from New Brunswick and Nova Scotia (Larson et al. 2000).

##### Agabus erytropterus

Taxon classificationAnimaliaColeopteraDytiscidae

(Say)

###### Note.

*Agabus erytropterus* is reported from eight specimens collected at five localities of Kings County (Table 2).

###### Habitat.

This species is usually associated with slowly flowing water but occurs in depositional areas with thick organic silt (Larson et al. 2000), which is exactly the type of habitats where it was found in Prince Edward Island (Table 1).

###### Distribution in the Maritime Ecozone.

*Agabus erytropterus* is also known from the neighboring Provinces of New Brunswick and Nova Scotia (Larson et al. 2000).

##### Agabus punctulatus

Taxon classificationAnimaliaColeopteraDytiscidae

Aubé

###### Note.

*Agabus punctulatus* is reported from nine specimens collected at one locality in Prince County (Table 2).

###### Habitat.

The most typical habitat of this species is flooded grass along the margin of vernal ponds in open grassland areas (Larson et al. 2000), which is similar to the habitat where *Agabus punctulatus* was found in Prince Edward Island (Table 1).

###### Distribution in the Maritime Ecozone.

This species is known also from the neighboring Maritime Provinces of New Brunswick and Nova Scotia (Larson et al. 2000).

##### Agabus semipunctatus

Taxon classificationAnimaliaColeopteraDytiscidae

(Kirby)

###### Note.

*Agabus semipunctatus* is reported from nine specimens from six localities of Kings and Prince Counties (Table 2).

###### Habitat.

*Agabus semipunctatus* most often occurs in *Sphagnum* bogs but can also be collected regularly from *Carex*-choked waters (Larson et al. 2000). In Prince Edward Island, this species was essentially found in similar type of habitats (Table 1).

###### Distribution in the Maritime Ecozone.

This species is also known from the neighboring Maritime Provinces of New Brunswick and Nova Scotia (Larson et al. 2000) and the Magdalen Islands (Alarie 2009).

##### Colymbetes paykulli

Taxon classificationAnimaliaColeopteraDytiscidae

Erichson

###### Note.

*Colymbetes paykulli* is reported from two specimens each collected in Queens and Kings Counties (Table 2).

###### Habitat.

These beetles are almost entirely restricted to the cold water of *Sphagnum* bogs. They occur most frequently in small pools with emergent *Carex*, or along the edges of bog-ring lakes in embayments and beaver runs, often or near the willow zone (Larson et al. 2000). The two specimens collected in Prince Edward Island were from a *Sphagnum* bog and a shallow pool covered with vegetation respectively (Table 1).

###### Distribution in the Maritime Ecozone.

This species is known also from the neighboring Maritime Provinces of New Brunswick and Nova Scotia (Larson et al. 2000).

##### Graphoderus liberus

Taxon classificationAnimaliaColeopteraDytiscidae

(Say)

###### Note.

*Graphoderus liberus* is reported from two specimens collected in Prince County (Table 2).

###### Habitat.

These beetles generally occur in boggy (often brown water) ponds and lakes. They are usually found at the edge of the encircling bog in embayments or beaver runs (Larson et al. 2000). The two specimens collected in Prince Edward Island were from *Sphagnum* bog ponds (Table 1).

###### Distribution in the Maritime Ecozone.

This species is known also from the neighboring Maritime Provinces of New Brunswick and Nova Scotia (Larson et al. 2000).

##### Hydrocolus paugus

Taxon classificationAnimaliaColeopteraDytiscidae

(Fall)

###### Note.

This species is reported from several specimens collected at various localities in each county of the province (Table 2).

###### Habitat.

These beetles occur among moss or dense emergent vegetation and debris along the margins of small pools, ponds and springs. They occur in peatland as well as in sites where the water surface is shaded and the water is cool (Larson et al. 2000). Specimens were collected in Prince Edward Island from a variety of lentic and lotic habitats (Table 1).

###### Distribution in the Maritime Ecozone.

This species is known also from the neighboring Maritime Provinces of New Brunswick and Nova Scotia (Larson et al. 2000), and the Magdalen Islands (Alarie 2009).

##### Hydroporus fuscipennis

Taxon classificationAnimaliaColeopteraDytiscidae

Schaum

###### Note.

This species is reported from several specimens collected at several localities of Prince and Kings Counties (Table 2).

###### Habitat.

These beetles occur among dense emergent vegetation along the margins of small, often temporary pools, usually situated in grasslands, cleared areas or at the edges of forest, but not typically in forest pools. *Hydroporus fuscipennis* is usually found in more or less eutrophic pools and is not common in peatlands (Larson et al. 2000). Specimens were collected in Prince Edward Island essentially from cattail ponds and roadside ditches (Table 1).

###### Distribution in the Maritime Ecozone.

This species is known also from the neighboring Maritime Provinces of New Brunswick (Larson et al. 2000).

##### Hydroporus gossei

Taxon classificationAnimaliaColeopteraDytiscidae

Larson & Roughley

###### Note.

This species is reported from several specimens collected in Prince and Kings Counties (Table 2).

###### Habitat.

In Newfoundland, this species has been collected from flooded grasses and emergent *Carex* along the margins of beaver ponds and roadside ponds. Specimens, which occurred on peaty substrates near boggy areas, were collected from areas exposed to the sun as well as from shorelines shaded by overhanging alder. In Prince Edward Island, *Hydroporus goosei* was collected in similar habitats, in addition to *Sphagnum* bogs and eutrophic creeks (Table 1).

###### Distribution in the Maritime Ecozone.

This large, distinctive *Hydroporus* Clairville species has generally been confused with *Hydroporus rectus* Fall. In the Maritime ecozone, *Hydroporus gossei* is also reported from the neighboring Province of New Brunswick (Larson et al. 2000).

##### Hydroporus obscurus

Taxon classificationAnimaliaColeopteraDytiscidae

Sturm

###### Note.

This species is reported from 16 specimens collected at two localities of Prince and Kings Counties (Table 2).

###### Habitat.

This species occurs in very small peatland pools, as well as in the moss mat along the margins of larger peatland pools and ponds (Larson et al. 2000). Specimens collected in Prince Edward Island were from a *Sphagnum* bog and the grassy shoreline of a pond (Table 1).

###### Distribution in the Maritime Ecozone.

This species is known also from the neighboring Maritime Provinces of New Brunswick and Nova Scotia (Larson et al. 2000).

##### Hydroporus striola

Taxon classificationAnimaliaColeopteraDytiscidae

(Gyllenhal)

###### Note.

*Hydroporus striola* was one of the most abundant (4.25%) and most common species collected in the province (Table 2).

###### Habitat.

*Hydroporus striola* occurs in almost all types of small, standing water where emergent vegetation is dense. Specimens occur in peatland pools, being most common in fen habitat and rather infrequent in ombrotrophic bog pools (Larson et al. 2000). Specimens collected in Prince Edward Island were from an array of lentic habitats as described above (Table 1).

###### Distribution in the Maritime Ecozone.

*Hydroporus striola* is the most ubiquitous species of *Hydroporus* in the boreal zone of the North Temperate Region (Larson et al. 2000). In the Maritime ecozone, this species is known also from the neighboring Provinces of New Brunswick and Nova Scotia (Larson et al. 2000), and the Magdalen Islands (Alarie 2009).

##### Hydroporus tenebrosus

Taxon classificationAnimaliaColeopteraDytiscidae

LeConte

###### Note.

Several specimens of *Hydroporus tenebrosus* were collected in each of the three counties of Prince Edward Island (Table 2).

###### Habitat.

This species occurs mainly in forested regions but is also common in the prairie parkland. Beetles occur in the emergent zone of small, usually temporary pools and ponds. They are usually found in eutrophic, sun-warmed sites (Larson et al. 2000). In Prince Edward Island, *Hydroporus tenebrosus* was almost essentially collected only in ponds, ephemeral pools, and roadside ditches (Table 1).

###### Distribution in the Maritime Ecozone.

The species is known also from the neighboring Provinces of New Brunswick and Nova Scotia (Larson et al. 2000), and the Magdalen Islands (Alarie 2009).

##### Hydroporus tristis

Taxon classificationAnimaliaColeopteraDytiscidae

(Paykull)

###### Note.

Several specimens of *Hydroporus tristis* were collected from Kings and Prince Counties (Table 2).

###### Habitat.

This species occurs in a variety of lentic habitats. Specimens are usually found among dense emergent vegetation at the margin of small, often more or less shaded pools. *Hydroporus tristis* is also common in small peatland pools (Larson et al. 2000). Whereas collected in few eutrophic creeks in Prince Edward Island, *Hydroporus tristis* was most generally found in ponds and shallow pools characterized by heavy accumulation of organic debris as well as in *Sphagnum* bogs (Table 1).

###### Distribution in the Maritime Ecozone.

The species is known also from the neighboring Province of New Brunswick (Larson et al. 2000), and the Magdalen Islands (Alarie 2009).

##### Hygrotus compar

Taxon classificationAnimaliaColeopteraDytiscidae

Fall

###### Note.

*Hygrotus compar* is reported from six specimens collected Prince County (Table 2).

###### Habitat.

In the Prairies, *Hygrotus compar* has been recorded from the margin of temporary ponds, usually in fresh water but specimens have also been found in saline ponds (Larson et al. 2000). In Prince Edward Island, specimens were found in a roadside ditch and a clay bed pond at the edge of a spruce and aspen forest (Table 1).

###### Distribution in the Maritime Ecozone.

The species is known also from the neighboring Province of New Brunswick (Larson et al. 2000).

##### Hygrotus laccophilinus

Taxon classificationAnimaliaColeopteraDytiscidae

(LeConte)

###### Note.

*Hygrotus laccophilinus* is reported from 22 specimens collected throughout the province (Table 2).

###### Habitat.

This species has been collected from a variety of small ponds. It appears to be most common among emergent grasses and sedges along the margin of permanent, or at least long lasting ponds. Specimens have been found most frequently in ponds in open country cleared of forest but they have also been collected in woodland ponds, especially beaver ponds (Larson et al. 2000). In Prince Edward Island, specimens were most frequently found in ponds and roadside ditches (Table 1).

###### Distribution in the Maritime Ecozone.

The species is known also from the neighboring Provinces of New Brunswick and Nova Scotia (Larson et al. 2000).

##### Ilybius discedens

Taxon classificationAnimaliaColeopteraDytiscidae

Sharp

###### Note.

Although found in relatively low numbers (seven specimens), this species is reported from each of the three Prince Edward Island counties (Table 2).

###### Habitat.

*Ilybius discedens* is one of the most characteristic water beetle species of boreal peatland generally occurring in small, moss-ringed pools, often where the water is cold to the touch (Larson et al. 2000). In Prince Edward Island, the majority of specimens were collected from *Sphagnum* bogs, although a few specimens were associated with eutrophic lotic habitats (Table 1).

###### Distribution in the Maritime Ecozone.

This species is also known from the neighboring Provinces of New Brunswick and Nova Scotia (Larson et al. 2000), and the Magdalen Islands (Alarie 2009).

##### Ilybius erichsoni

Taxon classificationAnimaliaColeopteraDytiscidae

(Gemminger & Harold)

###### Note.

This species is reported from three localities of Prince County (Table 2).

###### Habitat.

This is a species of forested regions where it occurs amongst dense vegetation, usually *Carex*, at the margins of both temporary and permanent ponds (Larson et al. 2000). Except for one specimen, which was collected in a shallow creek, all specimens of *Ilybius erichsoni* were collected in the type of habitats mentioned by Larson et al. (2000) (Table 1).

###### Distribution in the Maritime Ecozone.

This species is known also from the neighboring Provinces of New Brunswick and Nova Scotia (Larson et al. 2000), and the Magdalen Islands (Alarie 2009).

##### Ilybius larsoni

Taxon classificationAnimaliaColeopteraDytiscidae

(Fery & Nilsson)

###### Note.

This species is reported from five localities of Queens and Kings Counties (Table 2).

###### Habitat.

Specimens of *Ilybius larsoni* have generally been collected from small, cold woodland pools, usually with moss and accumulation of plant debris such as grass or sedge stalks or fallen leaves. Specimens have also been collected from small, peat-rich springs (Larson et al. 2000). In Prince Edward Island, this species was found in *Sphagnum* bogs and eutrophic creek (Table 1).

###### Distribution in the Maritime Ecozone.

This species is known also from the neighboring Provinces of New Brunswick and Nova Scotia (Larson et al. 2000).

##### Laccornis latens

Taxon classificationAnimaliaColeopteraDytiscidae

(Fall)

###### Note.

*Laccornis latens* is reported from two specimens collected in Kings County (Table 2).

###### Habitat.

The most common habitat for this species is semi-permanent, cool, shaded pools with deep leaf debris but few vascular plants; moss is often abundant (Larson et al. 2000). In Prince Edward Island, the two specimens sampled were collected within shoreline moss in a fen densely covered with *Scirpus* (Table 1).

###### Distribution in the Maritime Ecozone.

This species is known also from the neighboring Provinces of New Brunswick and Nova Scotia (Larson et al. 2000).

##### Neoporus carolinus

Taxon classificationAnimaliaColeopteraDytiscidae

(Fall)

###### Note.

*Neoporus carolinus* is reported from several specimens collected in Prince and Queens Counties (Table 2).

###### Habitat.

This species occurs among emergent vegetation such as sedges (Larson et al. 2000). In Prince Edward Island, beetles were collected from both lotic and lentic habitats, most of which characterized by dark brown water (Table 1).

###### Distribution in the Maritime Ecozone.

*Neoporus carolinus* is known also from the neighboring Provinces of New Brunswick and Nova Scotia (Larson et al. 2000).

##### Neoporus clypealis

Taxon classificationAnimaliaColeopteraDytiscidae

(Sharp)

###### Note.

*Neoporus clypealis* is reported from 22 specimens collected in each of the three Prince Edward Island counties (Table 2).

###### Habitat.

This species occurs among emergent vegetation such as sedges, along the margins of slow, marshy streams, beaver ponds, and small lakes. Beetles are generally found where there is some water movement and are usually on mineral substrates (Larson et al. 2000). With few exceptions, most specimens collected in Prince Edward Island were from creeks and small rivers (Table 1).

###### Distribution in the Maritime Ecozone.

*Neoporus clypealis* is known also from the neighboring Provinces of New Brunswick and Nova Scotia (Larson et al. 2000).

##### Neoporus sulcipennis

Taxon classificationAnimaliaColeopteraDytiscidae

(Fall)

###### Note.

Several specimens of *Neoporus sulcipennis* were collected at two localities of Prince County (Table 2).

###### Habitat.

This species occurs in small to medium sized warm, clear streams, often in depositional areas along the stream margins (Larson et al. 2000). In Prince Edward Island, all specimens were collected along the margins of rivers (Table 1).

###### Distribution in the Maritime Ecozone.

*Neoporus sulcipennis* is known also from the neighboring Province of New Brunswick (Larson et al. 2000). The presence of this species in Prince Edward Island represents the easternmost record in Canada.

##### Rhantus consimilis

Taxon classificationAnimaliaColeopteraDytiscidae

Motschulsky

###### Note.

*Rhantus consimillis* is reported from five specimens collected at the same locality of Prince County (Table 2).

###### Habitat.

This species occurs in warm, weedy ponds, generally in open grassland areas. It has been collected from both permanent and temporary habitats (Larson et al. 2000). The specimens collected in Prince Edward Island were from a permanent pond located at the edge of a spruce and willow forest (Table 1).

###### Distribution in the Maritime Ecozone.

Prior to this study, *Rhantus consimilis* had only been reported in New Brunswick (Webster 2008). The presence of this species in Prince Edward Island is its easternmost report in Canada.

##### Rhantus sinuatus

Taxon classificationAnimaliaColeopteraDytiscidae

(LeConte)

###### Note.

Five specimens of *Rhantus sinuatus* were collected at two localities of Kings County (Table 2).

###### Habitat.

This species occurs in lentic habitats densely overgrown with emergent vegetation. The species is common in bogs, but it also occurs in habitats with dense sedges, rushes or *Typha* (Larson et al. 2000). In Prince Edward Island, specimens were collected from a permanent pond with *Nymphaea* and a *Sphagnum* bog (Table 1).

###### Distribution in the Maritime Ecozone.

*Rhantus sinuatus* is known also from the neighboring Provinces of New Brunswick and Nova Scotia (Larson et al. 2000).

##### Rhantus suturellus

Taxon classificationAnimaliaColeopteraDytiscidae

(Harris)

###### Note.

One specimen of *Rhantus suturellus* was collected in Kings County (Table 2).

###### Habitat.

These beetles occur in cold, densely shaded water in forested areas, frequently found in bogs and fens (Larson et al. 2000). In Prince Edward Island, the specimen collected was found in a *Carex* pool with black sediments in a spruce forest (Table 1).

###### Distribution in the Maritime Ecozone.

*Rhantus suturellus* is known also from the neighboring Provinces of New Brunswick (Webster 2008) and Nova Scotia (Larson et al. 2000) and the Magdalen Islands (Alarie 2009).

##### Sanfilippodytes pseudovilis

Taxon classificationAnimaliaColeopteraDytiscidae

(Young)

###### Note.

One specimen of *Sanfillipodytes pseudovilis* was collected in Prince County (Table 2).

###### Habitat.

The species is very common in cold stenothermal springs where it can be taken either in the limnocrene pools or among the mosses along the spring margin (Larson et al. 2000). In Prince Edward Island, the only specimen collected was found in a narrow and shallow creek flowing on a sandy bed (Table 1).

###### Distribution in the Maritime Ecozone.

This species is also known from the neighboring Province of New Brunswick (Webster 2008) and the Magdalen Islands (Alarie 2009).

## Discussion

A total of 79 Hydradephaga species was recovered from 98 samples during a survey conducted on Prince Edward Island, Canada, between 2004–2005. Included among these were 30 new provincial records consisting of one haliplid, 2 gyrinids and 27 dytiscids (Table 2). *Acilius sylvanus*, *Rhantus consimilis* and *Neoporus sulcipennis* stand out as representing the easternmost reports of these species in Canada. In addition to these new records, one species, *Gyrinus aquiris* (Gyrinidae), which had been removed by Majka (2008), is reinstated in the faunal list of Prince Edward Island. According to this study and literature, 84 species of Hydradephaga are currently known from Prince Edward Island (Table 3). There are records of 67 species from Queens County, 66 from Kings County and 58 from Prince County.

The Nearctic component of the fauna of Prince Edward Island is made up of 68 species (80.9%), the Holarctic component of 16 species (19.1%). Most species are characteristic of both the Boreal and Atlantic Maritime Ecozones and have a transcontinental distribution, except for *Acilius mediatus* (Say), *Acilius sylvanus*, *Agabus erytropterus*, *Agabus subfuscatus* Sharp, *Copelatus glyphicus* (Say), *Dineutus nigrior* Roberts, *Dytiscus verticalis* Say, *Ilybius biguttulus* (Germar), *Ilybius larsoni*, *Gyrinus lecontei* (Hope), *Haliplus connexus*, *Hydroporus gossei*, *Hydroporus niger* Say, *Laccophilus maculosus maculosus* Say, *Laccornis latens*, *Liodessus affinis* (Say), *Nebrioporus rotundatus* (LeConte), *Neoporus carolinus*, *Neoporus clypealis*, and *Neoporus sulcipennis*, which are generally recognized as species with eastern affinities (Larson et al. 2000; Bousquet et al. 2013).

The composition of the Prince Edward Island fauna reflects that of the Maritime Provinces as a whole. All the species found on the island have also been recorded in New Brunswick (Webster 2016) and all but 13 [*Dineutus assimilis* Kirby, *Haliplus canadensis* Wallis, *Peltodytes tortulosus* Roberts, *Acilius sylvanus*, *Copelatus glyphicus*, *Hydroporus fuscipennis*, *Hydroporus gossei*, *Hydroporus tristis*, *Hygrotus compar*, *Hygrotus turbidus* (LeConte), *Neoporus sulcipennis*, *Rhantus consimilis*, and *Sanfilippodytes pseudovilis*] have also been recorded in Nova Scotia. The absence of these 13 species in the latter province is possibly attributable to a lack of collection effort.

In an examination of the Hydradephaga of insular portions of Atlantic Canada (Table 4), we found that despite significantly different land areas, and different distances to the neighbouring continental mainland, the island faunas of Prince Edward Island (with a land area of 5,660 km^2^ and 13 km from the mainland) and insular Newfoundland (with a land area of 111,390 km^2^, 18 km distant from Labrador and 110 km from Cape Breton Island) are very similar in the number of species (84 and 94 species respectively) despite differences in composition. The fauna of Cape Breton Island, however, (with a land area of 10,311 km^2^ and 1.5 km from the mainland) was 39% less diverse than that of Prince Edward Island consisting of 53 species. In view of the results obtained in this survey, this difference likely could be due to the comparative lack of collecting efforts on Cape Breton Island. In that regard, results from a similar field oriented research on the biodiversity of Hydradephaga of Cape Breton Island (Alarie, in prep.), should add to our knowledge on the faunistic composition of the group in this region.

**Table 4.** Comparison of total number of Hydradephaga species by family in the Maritime Ecozone.

	NB	NS	CBI	PEI	SI	MI	NF
Gyrinidae	19	20	9	12	0	7	10
Haliplidae	14	12	4	7	1	2	4
Dytiscidae	108	89	38	65	9	44	80
Total	141	121	51	84	10	53	94

**Notes**: NB , New Brunswick ; NS , Nova Scotia ; CBI , Cape Breton Island ; PEI , Prince Edward Island ; SI , Sable Island ; MI , Magdalen Island Archipelago ; NF , insular Newfoundland . Information is derived from Larson et al. (2000), Alarie (2009), Majka (2008), Webster (2016), this paper. Information from Newfoundland is provided as a basis of comparison.

## Conclusions

At the light of the many additions made to the faunal list of Prince Edward Island, the preceding account clearly represents a thorough treatment of the aquatic Adephaga of the province. The extensive field oriented research conducted on the island helps at providing detailed distribution of the Hydradephaga species in this province as well as detailed habitat information. Whereas additional species could potentially be found, this paper is deemed to represent an accurate account of the faunistic diversity of Hydradephaga on Prince Edward Island.

## Supplementary Material

XML Treatment for Gyrinus aquiris

XML Treatment for Gyrinus latilimbus

XML Treatment for Gyrinus pugionis

XML Treatment for Haliplus connexus

XML Treatment for Acilius sylvanus

XML Treatment for Agabus bifarius

XML Treatment for Agabus erytropterus

XML Treatment for Agabus punctulatus

XML Treatment for Agabus semipunctatus

XML Treatment for Colymbetes paykulli

XML Treatment for Graphoderus liberus

XML Treatment for Hydrocolus paugus

XML Treatment for Hydroporus fuscipennis

XML Treatment for Hydroporus gossei

XML Treatment for Hydroporus obscurus

XML Treatment for Hydroporus striola

XML Treatment for Hydroporus tenebrosus

XML Treatment for Hydroporus tristis

XML Treatment for Hygrotus compar

XML Treatment for Hygrotus laccophilinus

XML Treatment for Ilybius discedens

XML Treatment for Ilybius erichsoni

XML Treatment for Ilybius larsoni

XML Treatment for Laccornis latens

XML Treatment for Neoporus carolinus

XML Treatment for Neoporus clypealis

XML Treatment for Neoporus sulcipennis

XML Treatment for Rhantus consimilis

XML Treatment for Rhantus sinuatus

XML Treatment for Rhantus suturellus

XML Treatment for Sanfilippodytes pseudovilis
